# Refractory solitary cervical lymph node metastasis after esophageal squamous cell carcinoma surgery and its successful treatment with immune checkpoint inhibitor: A case report and literature review

**DOI:** 10.1097/MD.0000000000019440

**Published:** 2020-03-06

**Authors:** Wenjing Song, Helei Wang, Yuanyuan Tian, Shiwei Liu, Xiao Chen, Jiuwei Cui, Yuguang Zhao

**Affiliations:** a Cancer Center; b Department of Gastrointestinal Surgery; c Department of Bone and Joint Surgery, the First Hospital of Jilin University, Changchun, Jilin, China.

**Keywords:** clinical whole exon sequencing, esophageal squamous cell carcinoma, immune checkpoint inhibitor, programmed cell death-ligand 1, tumor mutational burden

## Abstract

**Rationale:**

Although the early detection and treatment of non-metastatic esophageal cancer has improved, these patients’ prognoses are still poor. Most patients with radical treatment for esophageal cancer will relapse in 3 years, and the best treatment strategy after recurrence has not been uniformly accepted. Multiform treatments may be beneficial to recurrent patients.

**Patient concerns:**

A 60-year-old male patient, due to routinely health examination, ulcerated lesions 30 cm away from the incisors were found by gastroscopy, pathology showed esophageal squamous cell carcinoma (ESCC).

**Diagnosis:**

Due to the patient's pathology, he was diagnosed with ESCC.

**Interventions:**

The patient underwent radical surgery for ESCC on June 28, 2015. The left cervical lymph node metastasis occurred after 20 months, and lymph node metastasis carcinoma resection was performed. After that, concurrent chemoradiotherapy was implemented, 40 days after the end of the 4 courses of chemotherapy, the left cervical metastatic lymph nodes relapsed, radioactive particle implantation was carried out, and progressed again after 1 month. The patient took apatinib for 1 week but could not tolerate due to hand-foot syndrome. Immune checkpoint inhibitor (ICI) was administered since October 27, 2017.

**Outcomes:**

The therapeutic effect of immune checkpoint inhibitor was evaluated as partial response (PR) after 6 courses of treatment and complete response (CR) after 15 courses of treatment. To our knowledge, this is the first case report of successful immunotherapy for refractory esophageal squamous cell carcinoma.

**Lessons:**

The emergence of ICIs promotes the treatment of esophageal cancer to a new era. Our observations suggest that patients for whom schedule to receive anti-programmed cell death protein-1 (anti-PD-1)/programmed cell death-ligand 1 (PD-L1) immunotherapy may require genomic testing to predict whether tumors respond to ICIs. In this case, we also present the predictors for the efficacy of targeted immunotherapy. At present, no matter which predictor of PD-L1 expression, tumor mutational burden (TMB), microsatellite instability (MSI), and tumor-infiltrating lymphocyte (TIL), a single predictor may be unconvincing and cannot accurately estimate the efficacy of immunotherapy. Multiplex detecting methods and combined biomarkers may provide new strategies. Consensus need to be reached in order to be widely applied in future studies.

## 1 Introduction

**Figure 1:**
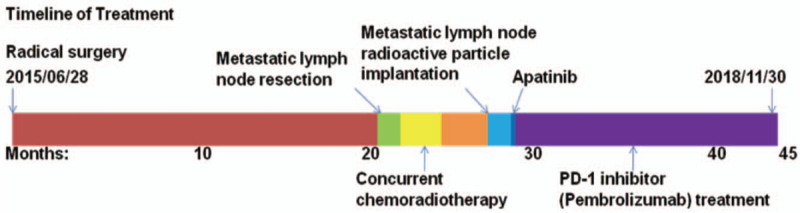
Timeline of treatment. Notes: The patient was diagnosed as esophageal squamous cell carcinoma and experienced radical surgery on June 28, 2015. As is depicted in the picture, the dark red color region represents the period from radical surgery to the discovery of metastatic lymph nodes in the left neck. The green region represents the period from metastatic lymph nodes dissection to the discovery of lymph node recurred of the left neck. The yellow region represents the period during which the patient received concurrent chemoradiotherapy. The orange area represents the period from the end of chemoradiotherapy to the metastatic lymph node of left neck progressed. The sky blue area represents the period after the implantation of radioactive particles in the metastatic lymph nodes. The dark blue area represents the period during which the patient took apatinib. The purple color represents the period during which the patient received PD-1 inhibitor treatment. PD-1 = programmed cell death protein-1.

Esophageal cancer is the sixth leading cause of cancer-related mortality,^[1]^ and the eighth most common cancer worldwide.^[2]^ Esophageal squamous cell carcinoma (ESCC) and esophageal adenocarcinoma are 2 main types of esophageal cancer. In China, esophageal cancer is the fourth leading cause of cancer-related mortality,^[3]^ with ESCC accounting for >90% of esophageal cancer cases^[4]^ and having a poor outcome with a 5-year survival rate of only about 15% to 25%.^[5,6]^ Surgery is the main method for the treatment of esophageal cancer, but with poor results. The 5-year survival rate of postoperative survival of esophageal cancer was only 20% to 30%, mainly due to postoperative recurrence and metastasis. About 34% to 79% of patients with ESCC relapse after surgery,^[7]^ while the recurrence rate of adenocarcinoma is as high as 50%.^[8]^ Over the past decade, metastatic ESCC has been managed primarily with chemotherapy, such as fluorouracil, cisplatin, and taxanes.^[9,10]^ Despite improvements in the management and treatment of these patients, overall outcome remains poor. The poor prognosis of ESCC highlights the urgent need for improved therapies, especially novel therapeutic approaches.^[11]^ Recently, breakthroughs in immune checkpoint blockade have offered new therapeutic options for many malignancies.^[11]^ PD-1, also known as CD279, is a inhibitory receptor expressed on activated T and B cells, which normally function to dampen the immune response.^[12–15]^ PD-1 is engaged by ligands PD-L1 (B7-H1, CD274) and PD-L2 (B7-DC, CD273), which are expressed by tumor cells and infiltrating immune cells.^[14,16]^ PD-L1 is upregulated in a variety of tumor cells. It binds to PD-1 on T cells and inhibits T cell proliferation and activation, leaving T cells inactive. Immune checkpoint inhibitors (ICIs) block the interaction of PD-1 and PD-L1, enhance T cell recognition of tumor cells, and ultimately restore antitumor immunity. ICIs targeting the PD-1 or PD-L1 have been shown to be effective in the management of many solid tumors, such as melanoma, non-small cell lung cancer, renal cell carcinoma, and so on.^[17–19]^ However, there are few reports on the treatment of recurrent metastatic esophageal cancer. Here, we reported a case of refractory solitary cervical lymph node metastasis after ESCC surgery, and its successful treatment with PD-1 inhibitor (Fig. 1). Furthermore, we discuss possible factors that could possibly predict the benefit from ICIs.

## 2 Case presentation

**Figure 2:**
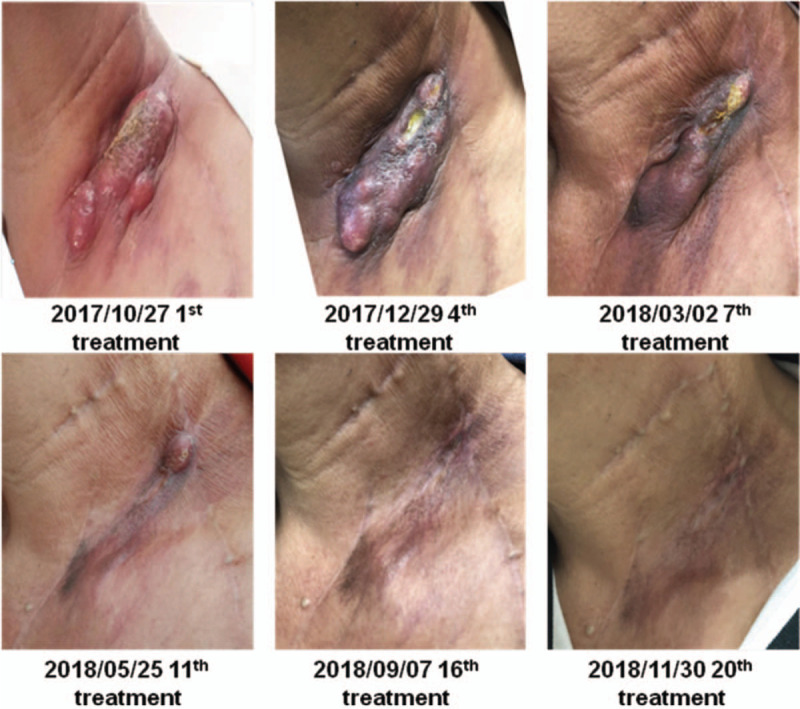
Therapeutic effect after PD-1 inhibitor treatment. Notes: The patient received PD-1 inhibitor (Pembrolizumab) treatment since October 27, 2017, the therapeutic effect was evaluated as PR after 6 courses of treatment and CR after 15 courses of treatment. The figures above showed the tumor changes. CR = complete response, PD-1 = programmed cell death protein-1, PR = partial response.

**Figure 3:**
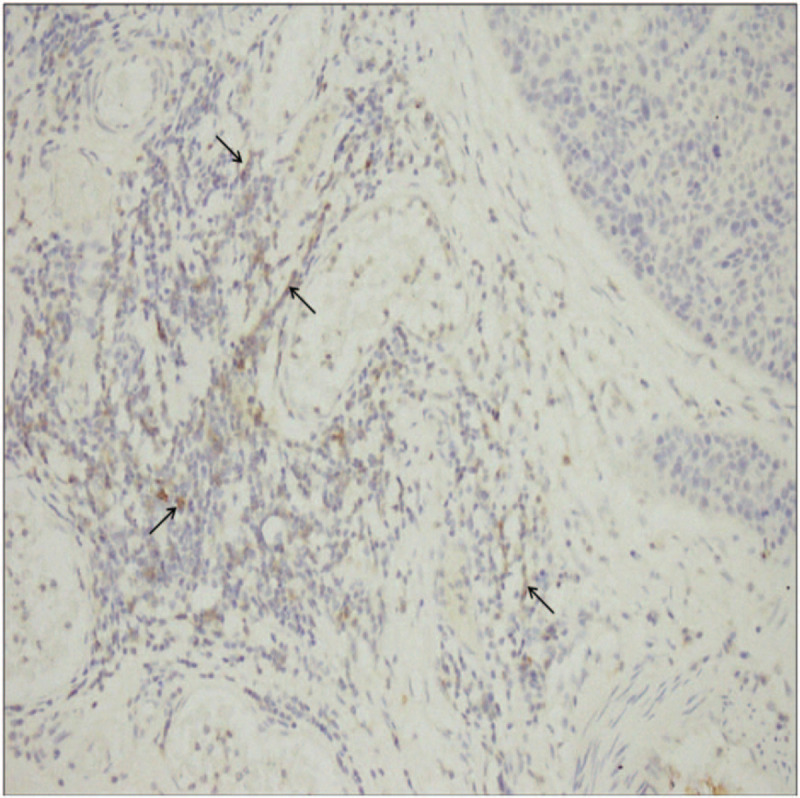
Lymphocyte infiltration in tumor tissues. Notes: At the upper right is the tumor tissue, with the arrow showing a large amount of lymphocyte infiltration around the tumor tissue (original magnification ×200).

**Figure 4:**
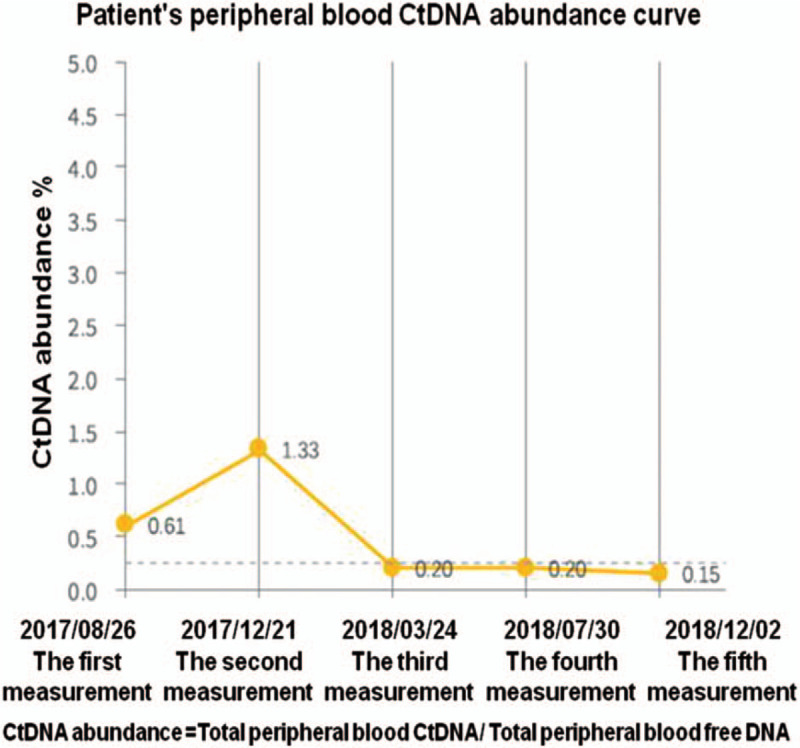
ctDNA abundance in peripheral blood before and after treatment with PD-1 inhibitor. Notes: The patient's peripheral blood circulating tumor DNA (ctDNA) abundance was measured for the first time on August 26, 2017, which was 0.61%. After the patient received three courses PD-1 inhibitor treatment, the peripheral blood ctDNA abundance was 1.33%, which was monitored on December 21, 2017, and decreased to 0.20% before the 8th treatment, which was monitored on March 24, 2018. The ctDNA abundance was still maintained at 0.20% on the fourth measurement, which was monitored before the 14th treatment, on July 30, 2018, and was further decreased to 0.15% after 20th treatment, which was monitored on December 2, 2018. PD-1 = programmed cell death protein-1.

A 60-year-old male patient, due to routinely health examination, ulcerated lesions 30 cm away from the incisors were found by gastroscopy, pathology showed ESCC, then performed radical surgery for esophageal cancer on June 28, 2015. Postoperative pathology showed that: moderately differentiated squamous cell carcinoma, invading the submucosa (tumor size: 1.8 × 1.5 × 0.4 cm), interstitial fibrosis of the cancer tissue, scattered focal lymphocytes, no involvement of vasculature and nerves, no lymph node metastasis (0/15). Among them, cardia lymph nodes (0/2), esophageal lymph nodes (0/13); immunohistochemistry (IHC): EGFR (+), P53 (80%+), Ki67 (70%+); Tumor Node Metastasis (TNM) staging was pT1bN0M0, stage I. Twenty months later, he unconsciously found mass on the left neck. Only left cervical lymph nodes (size: 4.7 × 3.7 cm) showed hypermetabolism by whole body Positron Emission Computed Tomography (PET-CT) (February 23, 2017), squamous cell carcinoma was verified by needle biopsy, so it was diagnosed as solitary left cervical lymph node metastasis, at this time, the TNM stage of the patient was cT0N0M1, stage IV. Metastatic lymph nodes were resected on March 5, 2017, and confirmed as squamous cell carcinoma by pathology; IHC: CK5/6 (local+), p40 (local+), CK7 (local+), CK20 (local+), P53 (70%+), Ki67 (50%+), EGFR (local+), CD56 (small focus+), CgA (–), Syn (–), EBER (–). Twenty days later, enlarged lymph nodes in the IV region of the left neck were found. According to the National Comprehensive Cancer Network (NCCN) guidelines, paclitaxel combined with platinum or docetaxel combined with platinum can be selected for locally advanced esophageal cancer. Therefore, we chose docetaxel combined with platinum. From March 28, 2017 to June 5, 2017, the patient received docetaxel (75 mg/m^2^) plus lobaplatin (25 mg/m^2^) for 4 courses of chemotherapy, simultaneously the patient received radiation therapy (50 Gy/25 f) from March 28, 2017 to May 3, 2017, the target area include the lymph node drainage area of the II to V region of the double neck and the esophageal anastomosis. After 2 courses of chemotherapy and 4 courses of chemotherapy, the efficacy was evaluated as stable disease (SD). After 40 days from the end of chemotherapy, the left cervical lymph nodes enlarged, and PET-CT showed no other metastasis. Squamous cell carcinoma was verified again by needle biopsy, and metastatic lymph node radioactive particle implantation was performed on August 18, 2017. After 1 month, the size of lymph nodes in the implanted area shrank, but the surrounding lymph nodes enlarged by comparison of PET-CT. After that, the patient took apatinib (500 mg qd) for only 1 week but could not tolerate due to hand-foot syndrome (CTCAE4.0 Grade III), which manifested as skin loss, ulcers, blisters in the palms and soles of the feet, and pain in the soles of the feet. We recommended that the patient took half the dosage, and the patient rejected the recommendation. Then the patient received PD-1 inhibitor (Pembrolizumab) treatment (200 mg, once every treatment, 21 days for a course of treatment) since October 27, 2017, the therapeutic effect was evaluated as PR after 6 courses of treatment and CR after 15 courses of treatment, as shown in Fig. 2. Only mild subclinical hypothyroidism was observed during the application, and the rest had no obvious side effects. There was also no dosage adjustment during the application. Before Pembrolizumab treatment, IHC indicated that PD-L1 (20%), MLH1 (+80%), MSH2 (+80%), MSH6 (+80%), from both the patient's postoperative specimens of esophageal primary lesion and metastatic lymph nodes. Results of esophageal primary lesion postoperative specimen detection by clinical whole exon sequencing (CWES): tumor mutational burden (TMB) value is 226. The number of insertion mutations and deletion mutations (Indels) is 58, and the ratio of Indels/TMB is 25.66%. Results of metastatic lymph nodes postoperative specimen detection by CWES: TMB is 203. The number of Indels is 29, and the ratio of Indels/TMB is 14.29%. In addition, lymphocyte infiltration in metastatic lymph nodes postoperative specimen is showed by HE staining (Fig. 3). Besides, the copy number of EGFR/CCND1/CKD4/CKD6/FGF3/FGF4/FGF19/MDM2/MDM4 in both postoperative specimens of esophageal primary lesion and metastatic lymph nodes by CWES are normal. The patient's peripheral blood circulating tumor DNA (ctDNA) abundance was monitored for the first time on August 26, 2017, which was 0.61%. After 7 courses PD-1 inhibitor treatment, the peripheral blood ctDNA abundance decreased to 0.20%, and further decreased to 0.15% after 20 courses of PD-1 inhibitor treatment, as shown in Fig. 4. To sum up, the patient benefited from PD-1 inhibitor treatment. Currently, the tumor has been controlled, the efficacy is CR, and the duration of response is 13.2 months.

## 3 Discussion

Despite recent advances in cancer treatment, the prognosis of esophageal cancer remains poor. The only potential curative treatment is surgical resection with adjuvant or neoadjuvant chemotherapy/chemoradiotherapy. Immune checkpoint blockade is a rapidly evolving treatment that has influenced the treatment guidelines for many tumor types. Esophageal cancer are tumors with high mutation loads that have attracted considerable attention since the beginning of interest in ICIs.^[20]^ In the clinical trial of KEYNOTE-028 and number ONO-4538-07/JapicCTI-No.142422, ICIs also showed promising antitumor activity and a manageable safety profile.^[21,22]^ Phase II clinical study KEYNOTE-180 further confirmed the sustained efficacy and controllable adverse effects of Pembrolizumab in the third-line and above treatment of advanced esophageal cancer.^[23]^ At the ASCO-GI conference on January 18, 2019, the KEYNOTE-181 study was published to establish the status of Pembrolizumab in the treatment of advanced esophageal cancer in larger sample sizes: compared with standard chemotherapy, Pembrolizumab can significantly prolong the overall survival of patients in the second-line treatment of PD-L1-positive (CPS [combined positive score] ≥10) advanced/metastatic esophageal cancer or esophagogastric adenocarcinoma patients. But not all patients benefit from these agents and several studies are trying to identify predictive and prognostic biomarkers to better understand and guide treatment decisions.^[24]^

Perhaps PD-L1 expression is the earliest and most widely recognized biomarker for predicting PD-1/PD-L1 blocking response. But PD-L1 expression has intratumoral heterogeneity. Moreover, PD-L1 expression is dynamic: it is expressed differently at different stages of the disease and can vary with treatment. Meanwhile, due to few studies on esophageal cancer, it remains controversial about the prognostic and predictive value of PD-L1 expression in ESCC, as some studies associate high PD-L1 expression with poor differentiation of tumor and poor prognosis, while others postulate better response to PD-1/PD-L1 blockade with high PD-L1 expression. Furthermore, cutoff value used for assessing PD-L1 expression may lack sensitivity and yield false-negative results,^[25]^ and currently there is no uniform criterion.

TMB as a new sensitive biomarker, has been demonstrated to be significantly associated with PD-1 and PDL-1 blocking response. Across different cancer types, cancers that have a higher TMB, thus a higher neoantigen exposure to the immune system, seem more likely to respond to ICIs.^[26–28]^ Although microsatellite instability (MSI) can result in high TMB, the role of TMB in predicting response to ICIs does not only include high levels of microsatellite instability (MSI-H). TMB represents the total number of tumor genome mutations in a given region. They are similar and different. MSI is a subgroup of TMB, while TMB represents a wider range of mutation maps, may be more widely used in the future. Genes associated with large increases in TMB include known DNA mismatch repair pathway genes (MSH2, MSH6, MLH1, PMS2) and DNA polymerases (POLE).^[29]^ Perturbations in mismatch repair gene expression, both loss and overexpression, can be deleterious to genomic stability,^[30–32]^ studies demonstrate alterations in the mismatch repair (MMR) pathway that lead to MSI-H.^[33]^ Clinically, mismatch repair deficient (dMMR) is often determined by immunohistochemistry of the 4 proteins of MLH1, MSH2, MSH6, and PMS2. Defects in DNA repair mechanisms and microsatellite instability /mismatch repair defects have emerged as potential useful clinical biomarkers.^[26,34]^ In addition to the common point mutations in cancer cells, insertion mutations, deletion mutations, together is Indels, result in the production of abnormal proteins, which increase the immunogenicity and thus activate the immune system. It showed that the number and percentage of Indels in renal cancer are the highest.^[35]^ This is the first scientific and reasonable explanation for why renal cancer is sensitive tumor to ICIs.

TILs is found to be an independent prognostic factor for prolonging progression free survival (PFS) and overall survival (OS) in tumor immunity,^[36]^ thus indicating the critical role of T cells in tumor immunity. In addition, ctDNA is now being extensively studied as it is a noninvasive “real-time” biomarker that can provide diagnostic and prognostic information before, during treatment and at progression.^[37]^ Therefore, “liquid biopsy” of ctDNA, may be an ideal one for patients with cancer.^[38]^

Although ICIs-induced pseudoprogression are a well-known phenomenon, hyperprogression has only recently been described.^[39–41]^ Some patients with MDM2 family amplification or EGFR aberrations had poor clinical outcome and significantly increased rate of tumor growth after single-agent ICIs (PD-1/PD-L1) treatment.^[42]^ In 2017, European Society for Medical Oncology (ESMO) conducted a comprehensive genetic analysis of patients with hyperprogression on immunotherapy, and found that MDM2/4 amplification, EGFR amplification, and CCND1/FGF3/FGF4/FGF19 amplification were associated with hyperprogression after ICIs treatment.^[42]^

In this patient, whether it is primary or metastatic specimens, PD-L1 expression is 20%, TMB values are both high, Indel number and percentage are also relatively high, high degree of lymphocyte infiltration, at the same time no hyperprogression factors. Combining all these indicators can predict the patient would response well to PD-1 inhibitors, but it is not realistic to clearly indicate that the patient would responde well to PD-1 inhibitors according to only 1 or 2 of these predictors. Fortunately, the patient's therapeutic effect confirms that the patient responded well to the PD-1 inhibitor. The monitoring data of ctDNA and clinical data show the effectiveness of its treatment. Dynamic monitoring shows that peripheral blood ctDNA was decreased. Although the ctDNA abundance was higher in the second measurement than the first, the peripheral blood ctDNA abundance was not measured at the beginning of PD-1 inhibitor treatment, the tumor was still proliferating during the period from August 26, 2017 to October 27, 2017. While the abundance of ctDNA was significantly reduced at the third measurement.

In summary, our observations suggest that patients for whom schedule to be received anti-PD-1/PD-L1 immunotherapy may require genomic testing to predict whether tumors respond to ICIs. Gene analysis may be a new approach for judging the potential clinical benefit of ICIs.^[43]^ Consensus need to be reached in order to be widely applied in future studies. At present, no matter which predictor of PD-L1 expression, TMB, MSI, and TIL, a single predictor may be unconvincing and cannot accurately estimate the efficacy of immunotherapy. Multiplex detecting methods and combined biomarkers may provide new strategies. Moreover, combination ICIs with existing chemotherapy or radiation or other immunotherapy with different mechanisms of action must be evaluated to achieve excellent outcomes in patients with ESCC. In the era of precision medicine, we still need to make efforts to improve the prognosis of this disease.

## 4 Conclusion

The treatment of recurrent refractory esophageal cancer was rarely reported. Although the surgical treatment, chemotherapy and radiotherapy are effective, the poor prognosis of esophageal cancer patients is still not improved. The emergence of ICIs pushes the treatment of esophageal cancer into a new era. However, there are still many unresolved issues that need to be further addressed. Future research directions may try to explore the utility of comprehensive assessments that take into account characteristics of the TMB and other immune parameters to produce a composite score predictive of benefit to ICIs.

## Author contributions

**Conceptualization:** Xiao Chen, Jiuwei Cui.

**Data curation:** Helei Wang, Shiwei Liu.

**Writing – original draft:** Wenjing Song, Yuanyuan Tian.

**Writing – review & editing:** Yuguang Zhao.

**Yuguang Zhao orcid:** 0000-0002-4430-7798.

**Wenjing Song orcid:** 0000-0003-2583-4362.

**Helei Wang orcid:** 0000-0001-5187-5112.

**Yuanyuan Tian orcid:** 0000-0001-9863-4498.

**Shiwei Liu orcid:** 0000-0003-0736-8628.

**Xiao Chen orcid:** 0000-0002-9043-1116.

**Jiuwei Cui orcid:** 0000-0001-6496-7550.

Yuguang Zhao orcid: 0000-0002-3274-0199.
